# Effect of Pterygium Removal Combined with Conjunctival Autograft on Corneal Parameters in Swept-Source Imaging

**DOI:** 10.3390/jcm11020329

**Published:** 2022-01-10

**Authors:** Marta P. Wiącek, Monika Kuśmierz-Wojtasik, Bogna Kowalska, Anna Machalińska

**Affiliations:** First Department of Ophthalmology, Pomeranian Medical University, 70-111 Szczecin, Poland; marta.p.wiacek@gmail.com (M.P.W.); m.d.kusmierz@gmail.com (M.K.-W.); bogna.kowalska96@gmail.com (B.K.)

**Keywords:** anterior segment OCT, astigmatism, conjunctival autograft, corneal tomography, corneal topography, fibrin tissue glue, high-order aberrations, pterygium, swept-source OCT

## Abstract

Background: Both pterygium ingrowth and excision determine alterations in corneal topography. The aim of this study was to evaluate the influence of pterygium removal combined with conjunctival autografts in addition to the use of human fibrin tissue glue on changes in corneal parameters as measured by 3-D swept-source anterior segment optical coherence tomography (AS-OCT) imaging. Methods: Sixteen eyes (16 patients) with pterygium that qualified for surgical treatment were enrolled in this study. Eye examination, slit lamp, and 3-D AS-OCT (CASIA 2) assessment were performed before the surgery and 7 days, 1 month, and 6 months after pterygium excision. Topographic parameters of both anterior and posterior surfaces of the cornea were analysed at each follow-up visit. Results: The gradual decrease in total astigmatism power from preoperative median 2.75 (6.15) D to 1.2 (1.1) D at 6-month follow-up (*p* = 0.034) was noted from the day 7 visit. Values were strongly influenced by variations of anterior cornea astigmatism. In contrast, a gradual total HOA reduction at the 1-month (from median 0.79 (1.3) D to 0.44 (0.27) D; *p* = 0.038) and at 6-month visits (0.25 (0.09); *p* = 0.001) was observed. Similarly, values were strongly influenced by variations of the anterior. Additionally, total average keratometry values increased from preoperative 44.05 (2.25) D to 44.6 (1.9) (*p* = 0.043) 1 month after the surgery. Conclusions: Significant steepening of the anterior cornea and a reduction in both astigmatism and HOA were observed after pterygium excision. The anterior corneal surface was an essential component of the total postoperative corneal topography values. Three-dimensional swept-source AS-OCT imaging seems to be a valuable tool for monitoring both the progression of the disease and postoperative effects in pterygium eyes.

## 1. Introduction

A pterygium is a degenerative lesion of the ocular conjunctiva characterized by the proliferation of fibrous-vascular tissue with its subsequent ingrowth into the cornea. The geographic variation in its incidence from 2% to 22% of the population has been described [1]. The progression of the lesion causes deterioration of the vision as a result of lesion ingrowth into the cornea, as well as a change in the curvature of the cornea or impaired refraction of the optical system of the eye [2,3]. Some studies have demonstrated that pterygia commonly induces focal corneal flattening and with-the-rule astigmatism. The putative causes of the observed topographic changes are mechanical distortion of the cornea due to traction forces in the horizontal meridian induced by the fibrous vascular membrane or indirect apparent flattening due to local tear film accumulation at the top of the pterygium [2,4].

The only effective method of therapy in pterygium is surgery [1,5]. Currently, the method of pterygium removal with autologous conjunctival transplantation plus the use of human fibrin tissue glue is one of the most innovative approaches, especially concerning low recurrence rate, reduced patient’s discomfort, and shortened recovery time after surgery with lack of serious complications [3,6]. However, little is still known about impact of this method on changes in corneal parameters after the procedure.

Since pterygium ingrowth results in stromal scarring and the cornea is covered by the lesion itself, the measurement of corneal topography and aberrations in both the anterior and posterior corneal surfaces may constitute a diagnostic problem in this disease. The novel three-dimensional (3-D) swept-source anterior segment optical coherence tomography (AS-OCT) provides high-resolution cross-sectional corneal images with increased penetration into opaque corneas [7,8]. Therefore, the usage of this device in corneas distorted by pterygium may provide substantial information about corneal topography. The aim of this study was to investigate the influence of autologous conjunctival autografts combined with the use of human fibrin tissue glue on changes in corneal parameters as measured by 3-D swept-source AS-OCT imaging.

## 2. Materials and Methods

### 2.1. Subjects

Sixteen eyes of sixteen patients with primary pterygium who met the following inclusion criteria were considered for the study:

- Persistent discomfort;
- Chronic inflammation;
- Visual impairment;
- Unfavorable cosmetic appearance.

The exclusion criteria were as follows:

- Diseases of the cornea that made it impossible to evaluate the impact of the surgery on corneal parameters (i.e., anterior segment disease other than pterygium, corneal scarring, and advanced cataracts);
- Previous ocular surgery within 6 months;
- The zero or first stage of pterygium.

**Figure 1:**
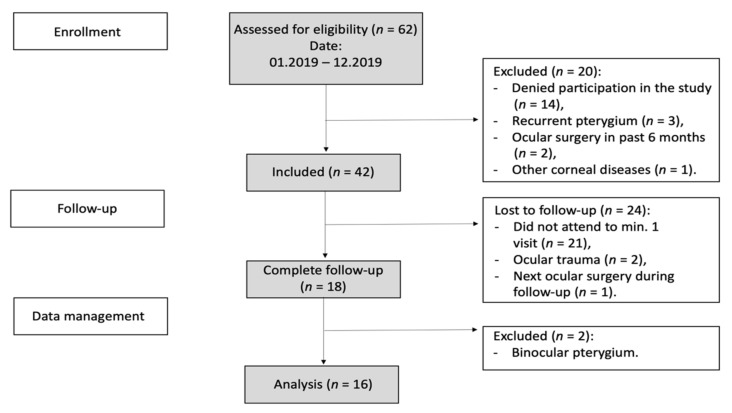
Flowchart of the study.

The study was designed as a prospective interventional study. The flowchart of the study is presented in Figure 1. Informed consent was obtained from each patient after explaining the nature of the study. This study was performed in accordance with the Declaration of Helsinki.

### 2.2. Clinical Examination

**Figure 2:**
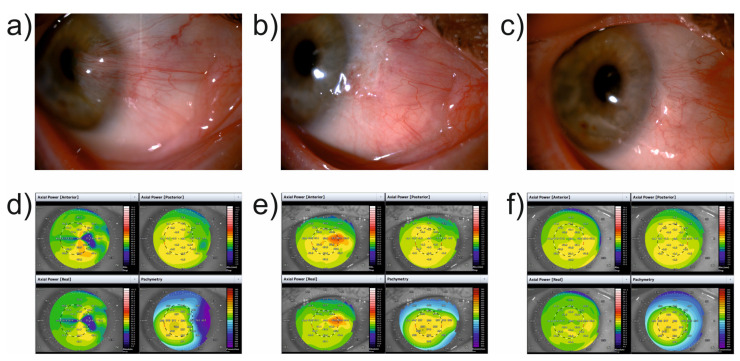
Anterior segment photography taken preoperatively at (**a**) 1 month (**b**) and 6 months (**c**) after pterygium removal by autologous conjunctival transplantation with human fibrin tissue glue; swept-source anterior segment optical coherence tomography taken preoperatively at (**d**) 1 month (**e**) and 6 months (**f**) post-surgery.

Ophthalmological examination was performed before the surgery, as well as 7 days and 1 and 6 months after the surgery. BCVA was measured at 5-meter distance on a Snellen letter chart. Each follow-up examination included examination of the anterior segment of the eyeball in the slit lamp with camera and 3-D swept-source AS-OCT (CASIA 2, Tomey Corporation, Nagoya, Japan), as presented in Figure 2. Slit lamp examination was aimed at assessing the severity of the disease based on a five-level scale: stage 0—pingueculum, posterior to the limbus; stage 1—the lesion involves the limbus with minimal papillary response and conjunctival and corneal tissues are flat; stage 2—the lesion appears just on the limbus with minimal elevation on the conjunctival and corneal tissues and normal vascularity; stage 3—the pterygium covers the area between the limbus and the papillary margin with moderate vascularity with vessel congestion and a lesion size up to 1 mm; stage 4—the lesion is central to the papillary margin and it extends for more than 1 mm with vessel congestion and dilation (10). During the entire observation period, AS-OCT measurements were taken by a trained operator, who also held the subjects’ eyelids, which was performed gently to avoid placing pressure on the globe. The measurements were obtained with the autoalignment function of the corneal topography map mode of the anterior segment ‘Corneal Topography’ module in ‘Corneal Map’ protocol. Consequently, 16 centered meridional scans with 800 A-scans per line were performed. During acquirement, the patient’s eye was fixated by the use of a dot target. Images were considered valid if the quality statement was not lower than 96.5%. Additionally, a minimum of 93% area within 6 mm of the central corneal area must be exposed for analysis. The images were analysed by built-in 2D analysis software version 3A.4 (Tomey Corporation, Nagoya, Japan) that automatically calculated measurements along with structural outlines and reference lines. The outline tracer was edited where needed. Central corneal thickness (CCT, μm), keratometry values (D), astigmatism power (D) and axis (°), astigmatism asymmetry (D), and high-order aberration power (HOA, D) were recorded and analysed at the indicated time points after surgery using the Fourier analysis 3D/2D function. The thickness of the cornea at its apex was assessed using the protocol with 16 radial cross-sections and a total scan time of 0.3 s. The values of the average keratometry (D) were calculated as a mean value of steep and flat meridian. Corneal astigmatism power (D) was expressed as the degree of astigmatism and the angle of astigmatism axis in 6 mm diameter. The astigmatism axis (°) was reported as an axial angle of the regular astigmatism component of corneal refractive power. Astigmatism asymmetry (D) was defined as a symmetric component of corneal refractive power in 6-millimeter diameter. Moreover, it corresponded to first-order component in Fourier analysis. Spherical equivalent (SE) was assessed as a component of corneal refractive power displayed as zero-order aberration in Fourier analysis. The second order component in the Fourier analysis was represented by regular corneal refractive power of astigmatism. Combined analyses of third or higher order components in Fourier analysis were termed as HOA power.

Measurements were read from both the anterior and posterior surfaces of the cornea, and the total topographic value of keratometry was taken into account. All values were assessed at a diameter of 6 mm. Only measurements that were well-centred and with high-quality indexes were included in the study. The spherical equivalent (SE, D) was calculated as a summation of the spherical refractive error and astigmatism power divided by two.

### 2.3. Surgical Technique

All procedures were carried out by the same surgeon (MW) using the same technique described as follows. After topical anesthesia with proxymetacaine, the planned excision site was marked. A solution of 2% lignokaine was injected between the conjunctiva and sclera. The pterygium head was removed by mechanical dissection from the cornea with the use of a raspatory, and the pterygium tail was cut off with surgical scissors. The surgical site was dried with a surgical sponge.

The conjunctival autograft transplantation site was marked. The conjunctiva was carefully dissected without disturbing the corneal limbus. The graft was transferred to cover the bare area of the sclera, and then it was stretched out over the operated site. The transplanted conjunctiva was attached to the operated site by applying the two components of human fibrin tissue glue, and the entire graft was compressed gently into position. Excessive human fibrin tissue glue was removed. Patients’ eyes were secured with a sterile dressing after instillation in the eyes of Maxitrol drops (suspension; 1 mL contains: 1 mg of dexamethasone, 3500 IU neomycin sulfate, and 6000 IU polymyxin B sulfate). The postoperative recommendation was a dosing of Maxitrol on the first day after surgery every two hours, then every 6 h for two weeks and every 12 h for the last two weeks.

### 2.4. Statistical Analysis

Statistical analysis was performed using Statistica 13.3 (TIBCO Software Inc., Palo Alto, CA, USA). Parametric variables were established by a Shapiro–Wilk test and by Levene’s test. Friedman’s repeated-measures analysis of variance (ANOVA) followed by Dunn–Bonferroni post hoc test was used to compare the different groups at consecutive evaluation time points. The Mann–Whitney U test was applied to evaluate the influence of pterygium staging on the corneal parameters. The correlations between the preoperative variables and corneal parameters were analysed with Spearman’s rank correlation coefficient (Rs). Data are presented as the mean ± SD unless stated otherwise according to value distribution. A *p* value of less than 0.05 was considered statistically significant. The number of 16 subjects followed in four consecutive time points was sufficient to detect, with 99% probability, the real effect size statistical power of the study at the 0.05 significance level. That corresponded to the minimal difference between the groups set at ±0.87 SD.

## 3. Results

Sixteen eyes of 10 males (62.5%) and 6 females (37.5%) were enrolled in the study. The mean age of the individuals was 59.72 ± 20.11 years (range from 23 to 85). In analyzed the group, two eyes (12.5%) represented stage 2 pterygium. Accordingly, 11 eyes (68.75%) were classified as stage 3, and three eyes (18.75%) were classified at stage 4 pterygium. Patients’ characteristics are presented in Table 1.

First, the influence of pterygium removal on BCVA improvement was evaluated. The preoperative BCVA was 0.64 ± 0.38, at 7-day visit it was 0.67 ± 0.36, and it increased further at the 1-month follow-up visit (0.70 ± 0.35) and at the 6-month follow-up examination (0.72 ± 0.36). However, statistical analysis did not reveal substantial changes in this parameter. This indicates that pterygium excision did not result in significant improvement of the function of the operated eyes.

A detailed summary of the dynamics of the changes in corneal keratometry values after pterygium removal accompanied by autologous conjunctival transplantation is presented in Table 2. Significantly increased values of total average keratometry were observed at 1-month follow-up (*p* = 0.043) in comparison to baseline values. This indicates that the traction forces were released due to the surgery, resulting in substantial steepening of the cornea in this follow-up point. In contrast, no changes in both anterior and posterior cornea values were detected. It is worth highlighting that the value of anterior surface keratometry in the steep meridian remained unchanged. This confirms that only the traction forces in the flat meridian were released as a result of pterygium removal. We noted a statistically significant positive correlation between the patient’s age and an improvement in average keratometry values of the total cornea 6 months after surgery (Rs = +0.60, *p* = 0.003). This confirmed that the release of traction forces due to surgery was more pronounced in older patients. Thus, the cornea is steeper as a consequence of pterygium removal in those individuals.

Since increasing astigmatism of the cornea is the leading complaint in patients with pterygium, this parameter was also evaluated. A significant reduction in total astigmatism power from the first month after the procedure with subsequent maintenance during the entire 6-month follow-up period was observed (Table 3). Interestingly, the mean axis of the baseline total astigmatism remained unchanged during the follow-up (*p* = 0.051). This means that as a consequence of pterygium removal, rotational symmetry of the cornea was restored. The follow-up analysis of the anterior corneal astigmatism dynamics revealed a similar decreasing pattern to variations in total corneal astigmatism observed as early as from the seventh day (Table 3). This highlights the impact of anterior corneal astigmatism changes on total corneal values. In contrast, significant changes in the power of posterior astigmatism were not noted. Moreover, a significant positive correlation between baseline total astigmatism and its reduction at the 6-month visit was observed (Rs: +0.78, *p* < 0.001). This means that in eyes with higher baseline values, a greater decrease in astigmatism after surgery was achieved.

Next, we evaluated irregular corneal astigmatism with asymmetry of the astigmatic components. The data are presented in Table 4. Interestingly, a significant reduction in total astigmatism asymmetry of the central 6-millimeter zone was observed at the 6-month follow-up visit (*p* = 0.049). According to the superficial location of the pterygium, the effect of its removal on the anterior surface of the cornea was established. Similarly, a significant reduction in astigmatism asymmetry of the anterior cornea in the central 6-millimeter diameter at the 6-month follow-up visit was noted (*p* = 0.049). However, at the corresponding follow-up visit, no significant difference in astigmatism asymmetry for the posterior cornea in the 6-millimeter zone was detected (*p* = 0.23).

Additionally, in order to perform a complex assessment of the impact of pterygium surgery on corneal topography, we analysed corneal HOA, which may influence vision acuity after the successful performance of corneal surgery. Gradual reductions in the central 6-millimeter HOA of the total cornea, as well as the anterior and posterior HOA after the treatment, were observed (Table 5). Interestingly, a significant reduction in both total and anterior surfaces HOA was detected beginning on the first month after pterygium surgery, while the values of posterior HOA did not decrease during the follow-up. We noticed significant positive correlations between baseline HOA values and their reduction at 6-month visits for full-thickness corneas (Rs: +0.94, *p* < 0.001), anterior (Rs: +0.93, *p* < 0.001), and posterior corneas (Rs: +0.69, *p* < 0.001). This observation indicated that in eyes with a higher baseline HOA, there was a greater reduction after pterygium surgery by autologous conjunctival autograft with human fibrin tissue glue. Accordingly, significant positive correlations between baseline astigmatism and changes in central HOA values 6 months after the procedure for total (Rs: +0.85, *p* < 0.001), anterior (Rs: +0.82, *p* < 0.001), and posterior (Rs: +0.49, *p* = 0.007) corneas were detected. We concluded that the higher the astigmatism value before pterygium surgery, the greater the reduction in HOA in all corneal layers.

Moreover, in comparison with baseline value, a substantial reduction in SE was noted at 1-month follow-up, 2.54 ± 1.43 D and 1.62 ± 0.78 D, respectively (*p* = 0.044). Similarly, 6 months after the treatment, a further decrease (mean ± SE: 0.42 ± 0.4 D, *p* = 0.01) was observed. This indicates that changes in corneal topography may consequently cause a significant reduction in refraction.

Additionally, in comparison with the baseline values, CCT decreased from the seventh day after the procedure (537.56 ± 38.56 µm preoperatively and 531.62 ± 37.22 µm on the 7th day). At 1-month and 6-month follow-up visits, the values were also reduced compared to the preoperative values (527.07 ± 33.44 µm and 532 ± 35.9 µm), but those differences did not show statistical significance (*p* = 0.101). This indicates that pterygium removal caused steepening of the cornea without its thinning.

Since pterygium may recur after surgical removal, in our group, we observed ingrowth of the conjunctiva crossing the border of the limbus in one eye (6.25%) at the 6-month visit. No side effects of the implemented treatment were observed.

## 4. Discussion

In this study, we evaluated the influence of pterygium removal accompanied by autologous conjunctival autografts and the use of human fibrin tissue glue on changes in corneal parameters, as measured by 3-D swept-source AS-OCT imaging. The superiority of the sutureless method in terms of shorter surgery duration and improvement of patient’s comfort in postoperative period without negative impact on complications rate has been well documented [9,10,11,12,13,14]. The location of the pterygium as a superficial corneal lesion determines its alterations of corneal topography. As a result of conjunctival ingrowth into corneal tissues, traction forces change corneal curvature, causing flattening of the horizontal meridian and, consequently, increasing topographic astigmatic aberration. Here, we documented a significant decrease in the power of total corneal astigmatism in response to pterygium removal during the 6-month follow-up time. Our results are in agreement with previous studies [2,4,6,15]. Additionally, in order to eliminate the possible occurrence of astigmatism associated with the procedure itself, an analysis of the astigmatism axis was performed. However, the axis did not change significantly during the 6-month follow-up. Hence, it can be concluded that the removal of the pterygium with the use of tissue glue did not induce this complication in the analysed group. Moreover, the postoperative decrease in the power of astigmatism seems to be a permanent phenomenon unrelated to surgically induced astigmatism. In comparison, Misra et al. [2] noted only a significant reduction in topographic astigmatism at the 3-month follow-up visit without substantial changes in subjective astigmatism after pterygium removal followed by conjunctival autograft with fibrin tissue glue. The putative influences of pterygium removal on the cornea were steepening of the cornea and decreased compression and distortion. To our knowledge, we are the first to observe that an increase in the patient’s age corresponds with greater postoperative steepening of the cornea. This may indicate that age-related weakening due to degeneration of the conjunctival elastotic fibres might be responsible for decreased traction forces after pterygium removal.

In comparison with bare sclera and amniotic membrane graft methods, a better astigmatic outcome in pterygium eyes operated on by the conjunctival autograft technique was previously reported [5,6]. Moreover, introduction of a tissue glue into the pterygium excision method has been described as leading to further significant improvements in surface regularity, as well as in the degree of corneal symmetry [2]. Accordingly, we observed a significant reduction in the asymmetrical component of astigmatism of the total and anterior cornea at the 6-month follow-up visit. It seems that the improvement in total corneal topography might be explained by the influence of the selected excision method on astigmatism values in the anterior cornea. Punjabi et al. [4] observed a significant reduction in astigmatism by performing a sutureless conjunctival autograft adhered by autologous blood. However, this observation was based on a short 6-week follow-up. Interestingly, the results of sutureless methods showed a greater reduction in total corneal astigmatism in cases of a more advanced stage of pterygium in a short-term follow-up [2,4]. Our study revealed that the higher the values of astigmatism the patient presented before the operation, the larger the decrease in this value. Importantly, our observation is based on a six-month follow-up visit, which allowed us to observe the dynamics of changes in the long term. It is worth emphasizing that a 6-month period after ocular surgery is sufficient for corneal curvature to remain stable over the long term and exhibits no further changes [16,17].

Patient satisfaction and final vision after surgery are strongly influenced by HOA. Due to the complex structure of the aberration, this problem cannot be solved by optical correction. In this study, a gradual reduction in total corneal HOA, as well as in both the anterior and posterior components, was observed. Similarly, Gumus et al. [18] reported a significant reduction in total corneal HOA at 12 months of observation. Increased pretreatment values of HOA might be associated with the pterygium size or a recurrent form of pterygium. Interestingly, we also noted that a higher baseline HOA corresponded to a larger decrease in HOA of all corneal compounds at 6-month follow-up. However, it was also pointed out that although most corneal distortions can be treated by surgical excision of the pterygium, some of them may not be completely removed due to the maintenance of irregular topographic changes of the cornea [15,18]. In comparison to our study, where a gradual decrease in HOA was observed during the entire 6-month follow-up time, Gumus et al. [18] reported no significant changes in HOA in the wavefront analyser between 3-month and 12-month values. However, none of the authors analysed the influence of corneal surfaces on total HOA. In our study, a strong impact of anterior HOA on the total HOA values was noted. Therefore, the inclusion of HOA analysis in our study provides a more comprehensive insight into the influence of pterygium excision on corneal parameters.

On the other hand, in our study, no significant changes in the astigmatism values of the posterior surface of the cornea during the entire 6-month follow-up were detected. This observation is consistent with other studies [17,19]. Contrary to our results, Levinger et al. [17] noted a substantial increase in the average keratometry of the posterior surface at the 3-month follow-up visit after pterygium removal using conjunctival autografts with tissue glue. The authors admitted that this might possibly be a result of surgically induced astigmatism.

Thus, in this study, we observed no substantial changes in lower-order aberrations expressed by astigmatism values of the posterior cornea in the analysed group. At the same time, no significant reduction in posterior HOA was detected. This confirms that pterygium removal combined with conjunctival autografting in addition to the use of human fibrin tissue glue has little impact on surgically induced aberrations of the posterior corneal surface.

It is worth mentioning that, to date, no studies have investigated changes in corneal parameters in the early postoperative period after pterygium removal by sutureless autologous conjunctival autografts, e.g., 7 days after surgery. Although Kam et al. [16] stated that cornea examination by bare sclera with the mitomycin C 0.02% method was performed on the seventh day visit after pterygium excision, baseline Scheimpflug-derived data acquisition was not possible in pterygium-affected eyes. This is a result of the data acquisition system based on a series of photos taken by a rotational camera from various angles in this device. The irregularity of a corneal shape that covers its surface by the ingrowth if the conjunctiva might constitute a limitation of this device in pterygium. Consequently, the authors presented only a comparative analysis of postoperative keratometry and astigmatism values instead of a preoperative and postoperative comparison. In contrast, in our study, we used 3-D swept-source AS-OCT and observed no acquisition limitations in the pterygium eyes. This might be considered an advantage of 3-D swept-source AS-OCT over Scheimpflug imaging. Additionally, the 3-D swept-source AS-OCTs provide analyses of universal usage in several corneal abnormalities, as well as ocular surgery and glaucoma. Consequently, that might result in greater availability of the device, especially in medical centres with a wide spectrum of patients. Moreover, a combined analysis of HOA without being split into particular components might be time saving and beneficial in daily practice. On the other hand, thanks to the introduction of an examination in the early postoperative period, we were able to assess the dynamics of changes in a more precise manner. Hence, after pterygium excision, a significant reduction in CCT from the seventh day after the surgery was observed. Accordingly, substantial changes in the anterior cornea expressed as decreases in both anterior astigmatism power and HOA were observed as early as 7 days and 1 month postsurgery. Consequently, the total value of astigmatism on the seventh day visit was significantly reduced. The significant decrease in values of the analysed parameters in the early postoperative period might indicate the minimally invasive character of autologous conjunctival autografts in addition to the use of human fibrin tissue glue.

In pterygium, a strong tendency for the disease to recur in the operated eye has been described [1,20,21,22,23]. Although the exact rate of recurrence is difficult to estimate, it strongly depends on the method of surgical removal. Accordingly, it may reach 88% in the case of bare sclera excision [23]. On the other hand, a recurrence rate of only 0.1% in pterygium extended removal followed by extended conjunctival transplant (PERFECT) was reported. However, an increased risk of severe complications with the PERFECT technique (i.e., corneal ulcers causing a loss of four lines of visual acuity, double vision, and severe pain), the long duration of the procedure, and the necessity of periocular or general anesthesia limit the indications for this procedure [3]. Nevertheless, it was proven that conjunctival autograft usage results in a significant decrease in the recurrence rate without an increased risk of severe adverse effects [20]. However, the impact of the graft fixation method (glue, suture, or both) on this phenomenon is unclear [3,24]. The recurrence rate in our study was established at 6.25%, which is in agreement with previous reports [23].

To the best of our knowledge, to date, none of the previous publications has analysed complex corneal parameters after pterygium surgery in order to extensively evaluate the values of the total cornea, as well as its particular anterior and posterior components. Although authors of this study put an effort to provide high quality data, some limitations might still be pointed out. A larger number of individuals could contribute to a more comprehensive analysis of the studied parameters. Moreover, a longer follow-up period might allow for better control of the dynamics of studied variables and could help to detect early features of the disease recurrence. Moreover, a greater representation of the particular stages of the pterygium might be beneficial for better detection of the relationship between disease advancement and changes of analysed parameters after the surgery. However, in this study, the sample was sufficient for determining the effect size of analysed variables. Additionally, since both Pentacam Scheimpflug imaging and 3-D swept-source AS-OCT are complementary methods in anterior segment evaluation, the comparison of utility and repeatability of both devices in eyes after pterygium surgery might be a field to explore in future studies.

## 5. Conclusions

As a result of pterygium surgery, we observed significant steepening of the cornea with a reduction in both astigmatic and HOA. Additionally, this study confirmed that the anterior corneal surface is an essential component of total postoperative astigmatism after pterygium excision. In summary, 3-D swept-source AS-OCT imaging seems to be a valuable tool for monitoring both the progression of the disease and postoperative effects on pterygium eyes.

## Figures and Tables

**Table 1:** Clinical characteristics of patients.

Parameter	Value
Age (years)	59.72 ± 20.11
Sex, n male/female	9/7
Affected eye, n (right/left)	9/7
Temporal/nasal, n	2/14
Preoperative BCVA (Snellen)	0.64 ± 0.38

Abbreviations: BCVA, best corrected visual acuity.

**Table 2:** Analysis of average keratometry in eyes with pterygium removal by autologous conjunctival autograft with human fibrin tissue glue.

Average Keratometry (D)	Preoperative	Day 7	1 Month	6 Months	*p* Value					
					BL vs. Day 7	BL vs. 1 Mo	BL vs. 6 Mo	day 7 vs. 1 Mo	day 7 vs. 6 Mo	1 Mo vs. 6 Mo
total	44.05 (2.25)	44.6 (2.9)	44.6 (1.9)	44.2 (1.8)	1	0.043	0.928	0.928	0.999	0.999
anterior	50.7 (3.14)	50.2 (4.55)	50.4 (2.7)	49.75 (0.7)	0.099	0.962	0.416	0.999	0.999	0.999
posterior	−6.4 (0.2)	−6.35 (0.25)	−6.2 (0.3)	−6.35 (0.1)	0.999	0.999	0.999	0.999	0.999	0.999

Friedman’s repeated-measures analysis of variance (ANOVA) and Dunn–Bonferroni post hoc test. Data were presented as median (interquartile range). Abbreviations: BL, baseline value; mo, month (s).

**Table 3:** Analysis of astigmatism power in eyes with pterygium removal by autologous conjunctival autograft with human fibrin tissue glue.

Astigmatism Power (D)	Preoperative	Day 7	1 Month	6 Months	*p* Value					
					BL vs. Day 7	BL vs. 1 Mo	BL vs. 6 Mo	Day 7 vs. 1 Mo	Day 7 vs. 6 Mo	1 Mo vs. 6 Mo
total	2.75 (6.15)	1.3 (1.9)	1 (1.4)	1.2 (1.1)	0.021	0.027	0.034	0.999	0.999	0.999
anterior	3.1 (6.85)	1.4 (2.05)	1.1 (1.6)	1.2 (0.6)	0.029	0.049	0.029	0.999	0.999	0.999
posterior	0.2 (0.15)	0.3 (0.15)	0.3 (0.1)	0.25 (0.2)	0.999	0.999	0.999	0.999	0.999	0.999

Friedman’s repeated-measures analysis of variance (ANOVA) and Dunn–Bonferroni post hoc test. Data were presented as median (interquartile range). Abbreviations: BL, baseline value; mo, month (s).

**Table 4:** Analysis of astigmatism asymmetry in central 6-millimeter zone in eyes with pterygium removal by autologous conjunctival autograft with human fibrin tissue glue.

Astigmatism Power (D)	Preoperative	Day 7	1 Month	6 Months	*p* Value					
					BL vs. Day 7	BL vs. 1 Mo	BL vs. 6 Mo	day 7 vs. 1 Mo	Day 7 vs. 6 Mo	1 Mo vs. 6 Mo
total	1.68 (1.79)	0.85 (1.86)	0.75 (1.05)	0.54 (0.42)	0.962	0.345	0.049	0.999	0.999	0.999
anterior	1.87 (2.0)	0.95 (2.06)	0.83 (1.17)	0.6 (0.46)	0.938	0.335	0.049	0.999	0.999	0.999
posterior	0.1 (0.19)	0.11 (0.1)	0.12 (0.16)	0.06 (0.08)	0.999	0.999	0.999	0.999	0.416	0.999

Friedman’s repeated-measures analysis of variance (ANOVA) and Dunn–Bonferroni post hoc test. Data were presented as median (interquartile range). Abbreviations: BL, baseline value; mo, month (s).

**Table 5:** Values of high order aberrations (D) in eyes with pterygium treated with autologous conjunctival transplantation with the use of human fibrin tissue glue.

Astigmatism Power (D)	Preoperative	Day 7	1 Month	6 Months	*p* Value					
					BL vs. Day 7	BL vs. 1 Mo	BL vs. 6 Mo	Day 7 vs. 1 Mo	Day 7 vs. 6 Mo	1 Mo vs. 6 Mo
total	0.79 (1.3)	0.53 (0.57)	0.44 (0.27)	0.25 (0.09)	0.823	0.038	0.001	0.999	0.155	0.999
anterior	0.88 (1.44)	0.59 (0.64)	0.49 (0.3)	0.28 (0.11)	0.592	0.029	0.001	0.999	0.191	0.999
posterior	0.05 (0.03)	0.04 (0.02)	0.03 (0.02)	0.03 (0.01)	0.999	0.823	0.592	0.125	0.079	0.999

Friedman’s repeated-measures analysis of variance (ANOVA) and Dunn–Bonferroni post hoc test. Data were presented as median (interquartile range). Abbreviations: BL, baseline value; mo, month (s).

## Data Availability

Data are available from the corresponding author upon request.
